# Presumed Cytokine-Mediated Mild Liver Injury With Similar Kinetics Following COVID-19, Acute Prostatitis, and mRNA Vaccination: A Case Report and Review of the Literature

**DOI:** 10.7759/cureus.104154

**Published:** 2026-02-23

**Authors:** Harukazu Hirano

**Affiliations:** 1 Department of Family Medicine, Koyo Seikyo Clinic, Fukui, JPN

**Keywords:** acute prostatitis, covid-19, cytokine-mediated injury, interleukin-6, liver injury, messenger rna vaccine, pro-inflammatory cytokine

## Abstract

Cytokine-mediated liver injury is well-documented in severe systemic inflammatory conditions, such as cytokine storms. However, its occurrence in mild inflammatory settings remains underrecognized and is rarely monitored longitudinally in a single individual. The author presents his own case involving recurrent episodes of transient, mild liver injury occurring in himself as a 72-year-old man. These episodes were temporally associated with three distinct inflammatory stimuli: COVID-19, acute prostatitis, and administration of a SARS-CoV-2 mRNA vaccine. Liver function tests performed during each event showed similar patterns of elevation followed by normalization. After mRNA vaccination, monitoring revealed a transient increase in proinflammatory cytokines, specifically IL-6 and tumor necrosis factor alpha, that coincided with the elevation of liver enzymes. The recurrence of this distinct pattern of mild liver injury suggests a potential host-specific predisposition to cytokine-mediated injury in response to various inflammatory stimuli. This observation warrants further investigation to clarify the pathophysiology and clinical significance of such mild reactions.

## Introduction

In primary care settings, liver injury associated with acute infection is occasionally observed. However, because it typically manifests as mild liver injury, its progression is rarely monitored in clinical practice. Mild liver injury often remains asymptomatic and clinically overlooked, yet its recognition is important for understanding an individual’s unique biological response to inflammation. In many patients, these transient elevations serve as a “subclinical window” into how their immune system interacts with various disease triggers. Identifying such patterns can help clinicians distinguish between routine physiological responses and host-specific predispositions to organ injury, which may have implications for personalized monitoring during infection or vaccination.

Liver injury linked to COVID-19 has been reported in mild cases [1]; however, most attention has focused on severe cases characterized by a cytokine storm (CS) [2]. Hepatic injury can result from several mechanisms, including direct damage from SARS-CoV-2, hypoxic-reperfusion injury, drug-induced liver injury (DILI) from medication toxicity, immune-mediated injury (in which the immune system attacks hepatocytes), and cytokine release [3]. Distinguishing among these mechanisms is essential, as clinical management and prognosis differ significantly. Similarly, hepatic injury due to bacterial infections, such as sepsis-induced liver injury, has been reported in severe cases and represents a classic example of CS [4]. However, liver injury associated with mild acute prostatitis has not been well documented. mRNA vaccines against SARS-CoV-2 have also been shown to increase cytokine levels [5], and although rare, subsequent liver injury, often immune-mediated, has been reported [6]. Despite these observations, the molecular mechanisms underlying cytokine-induced liver injury, particularly in mild inflammatory conditions, remain unclear.

Herein, the author presents a unique self-case report describing recurrent episodes of transient mild liver injury with similar kinetics. These episodes were characterized by consistent timing of enzyme peaks (three to six days post-stimulus), comparable magnitude of elevation (1.7- to 10-fold above baseline), and rapid normalization within seven to 28 days following three distinct inflammatory triggers: viral infection (COVID-19), bacterial infection (acute prostatitis), and mRNA vaccination. Through this longitudinal observation, the author aimed to highlight a potential common pathophysiology and underscore the need to investigate host-specific responses to mild inflammatory stimuli in future studies.

## Case presentation

This case study focuses on the author of this research. As a self-case report, it is important to acknowledge that subjective perception and reporting of symptoms may have been influenced by the author’s clinical background and personal involvement. To mitigate observer bias, objective laboratory data and chronological findings were prioritized to ensure reproducibility.

In January 2024, a 72-year-old male healthcare professional contracted COVID-19 (Episode 1), followed by an episode of acute prostatitis in May 2025 (Episode 2). Both episodes were associated with transient liver injury. Cytokine kinetics and liver function assessments were conducted before and after the administration of the eighth dose of the monovalent mRNA vaccine BNT162b2 (Pfizer-BioNTech) against SARS-CoV-2 in October 2025 (Episode 3). The patient was 170 cm tall, weighed 60 kg, and had a BMI of 20.8.

To manage hypertension and hyperlipidemia, he was prescribed daily doses of candesartan (8 mg), amlodipine (5 mg), and atorvastatin (10 mg). The patient had no prior history of liver disease, surgical interventions, or documented adverse reactions to nonsteroidal anti-inflammatory drugs or antibiotics. He was a nonsmoker and consumed ethanol equivalent to 14 g/day.

During biannual workplace health assessments conducted from 2014 to 2023, the following results were recorded: median aspartate aminotransferase (AST) was 25 U/L (IQR 23.3-28.0), median alanine aminotransferase (ALT) was 18.5 U/L (IQR 16.3-20.0), and median gamma-glutamyl transpeptidase (γ-GTP) was 73 U/L (IQR 66.5-81.5), occasionally showing slight elevations. All other test results, including hepatitis virus panels, were normal.

**Figure 1:**
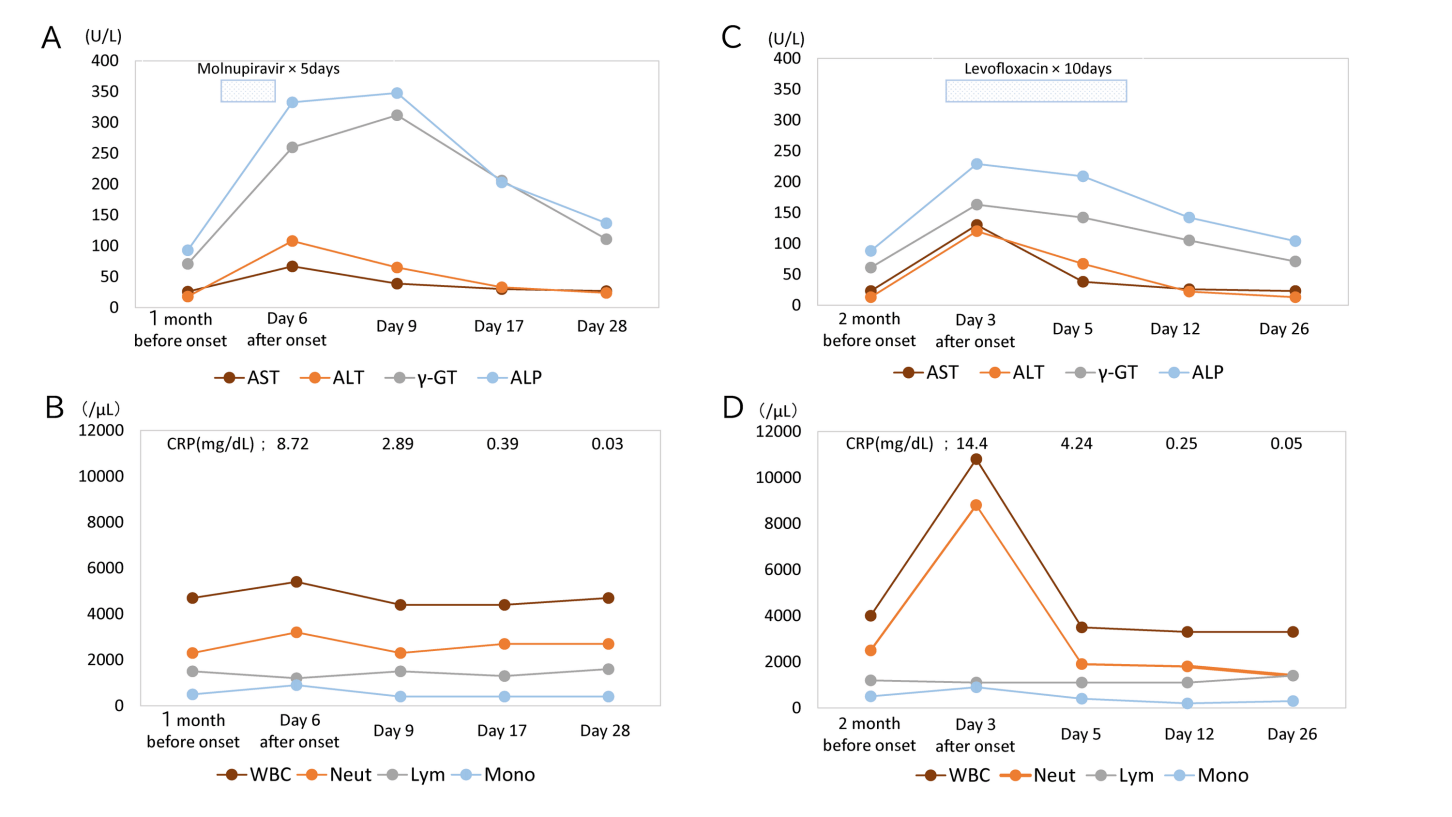
Kinetics of biomarkers associated with COVID-19 and acute prostatitis (A, B) Changes in liver function and peripheral blood parameters following the onset of COVID-19. (C, D) Changes in liver function and peripheral blood parameters following the onset of acute prostatitis. The parallel trends in enzyme elevation and subsequent normalization after both viral and bacterial stimuli suggest a consistent host-specific inflammatory response, regardless of the type of pathogen. ALP, alkaline phosphatase; ALT, alanine aminotransferase; AST, aspartate aminotransferase; γ-GTP, γ-glutamyl transpeptidase; Lym, lymphocyte; Mono, monocyte; Neut, neutrophil

In January 2024, the patient presented with fever (37.2°C), sore throat, cough, and fatigue (Episode 1). A SARS-CoV-2 antigen test returned positive, prompting treatment with molnupiravir (800 mg twice daily for five days) in combination with acetaminophen. Six days after symptom onset, blood tests revealed hepatic dysfunction: AST 67 U/L, ALT 108 U/L, ALP 333 U/L, and γ-GTP 260 U/L (Table 1, Table 2). These values represent increases of 5.4-, 3.7-, 3.6-, and 3.7-fold, respectively, compared with baseline. Symptoms improved within a week, and liver function normalized by days 17 and 28 (Figure 1). Investigations of immune competence in this case documented both humoral immune responses following COVID-19 infection and cellular immune responses after mRNA vaccination [7].

**Table 1:** Laboratory findings for the three episodes Comparison of peak laboratory values across the three episodes. Data represent the peak liver enzyme elevations for each event: six days after COVID-19 onset (Episode 1), three days after acute prostatitis onset (Episode 2), and three days following mRNA vaccination (Episode 3). Despite the different inflammatory triggers, the consistent timing of peak values and their subsequent normalization support a common cytokine-mediated mechanism. ALP, alkaline phosphatase; ALT, alanine aminotransferase; AST, aspartate aminotransferase; γ-GTP, γ-glutamyl transpeptidase; LDH, lactate dehydrogenase; TNF-α, tumor necrosis factor alpha

Laboratory value	Episode 1 (COVID-19), Day 6	Episode 2 (acute prostatitis), Day 3	Episode 3 (mRNA vaccination), Day 3	Reference range
Peripheral blood				
Red blood cells (×10⁴/μL)	442	453	464	438-577
Hemoglobin (g/dL)	14.6	14.9	15.1	13.6-18.3
Hematocrit (%)	44.2	44.2	45.4	40.4-51.9
Platelets (×10⁴/μL)	19.5	14.2	16.6	14.0-37.9
White blood cells (/μL)	5,400	10,800	3,800	3,500-9,700
Neutrophils (%/μL)	3,200 (58.9%)	8,800 (80.7%)	1,300 (43.6%)	42.0-74.0
Eosinophils (%/μL)	100 (1.1%)	0 (0%)	200 (6.6%)	0.0-7.0
Monocytes (%/μL)	900 (16.8%)	900 (8.5%)	300 (11.8%)	1.0-8.0
Lymphocytes (%/μL)	1,200 (22.9%)	1,100 (10.3%)	1,100 (36.9%)	18.0-50.0
Basophils (%/μL)	0 (0%)	0 (0%)	0 (0%)	0.0-2.0
Blood chemistry				
Total protein (g/dL)	7.1	6.8	-	6.6-8.1
Total bilirubin (mg/dL)	0.8	1.9	0.7	0.4-1.5
AST (U/L)	67	130	43	13-30
ALT (U/L)	108	120	39	10-42
LDH (U/L)	174	200	170	124-222
γ-GTP (U/L)	260	163	102	13-64
ALP (U/L)	333	229	-	38-113
Blood urea nitrogen (mg/dL)	13.6	16.5	18.3	8-20
Creatinine (mg/dL)	1.01	1.14	1.06	0.65-1.07
Immunology				
CRP (mg/dL)	8.72	14.41	0.69	0.0-0.14
Antinuclear antibody	-	Negative	-	0.0-1.0
Antimitochondrial M2 antibody	-	Negative	-	0.0-6.9
Anti-smooth muscle antibody	-	Negative	-	-
IL-6 (pg/mL)	-	-	5.52 (20 hours post-vaccination)	<2.59
TNF-α (pg/mL)	-	-	1.40 (20 hours post-vaccination)	<1.0
Urinalysis (qualitative)				
Protein	(-)	1 (+)	-	(-)
Glucose	(-)	1 (+)	-	(-)
Occult blood	(-)	2 (+)	-	(-)
Urobilinogen	Normal	Normal	-	Normal
Bilirubin	(-)	1 (+)	-	(-)
Ketones	(-)	1 (+)	-	(-)
Leukocytes	(-)	4 (+)	-	(-)
Urine microscopy				
Red blood cells	-	5-9	-	(-)
White blood cells	-	>100	-	(-)
Epithelial cells	-	<1	-	(-)
Bacteria	-	(-)	-	(-)

**Table 2:** Clinical summary and comparison of the three episodes of transient liver injury Comparison of clinical parameters across three distinct inflammatory triggers. Fold increases for each enzyme were calculated relative to the patient’s individual 10-year baseline median values. The consistent peak timing (within three to six days or ~20 hours post-stimulus) and rapid normalization of liver enzyme elevations were observed in all episodes, regardless of the severity of systemic symptoms. ALT, alanine aminotransferase; AST, aspartate aminotransferase; γ-GTP, γ-glutamyl transpeptidase; TNF-α, tumor necrosis factor alpha

Episode	Triggering event	Peak day (post-stimulus)	Peak AST/ALT (U/L)	Peak γ-GTP (U/L)	Measured cytokines	Resolution (days)
1	COVID-19 infection	Day 6	67/108	260	Not measured	28
2	Acute prostatitis	Day 3	130/120	163	Not measured	26
3	mRNA vaccination	Day 3	43/39	102	IL-6: 5.52 pg/mL/TNF-α: 1.40 pg/mL	7

In May 2025, the patient exhibited fever (37.3°C), fatigue, and urinary urgency (Episode 2). By the third day, laboratory results showed a white blood cell count of 10,800/µL with 80.7% neutrophils, and urinalysis demonstrated pyuria. At this time, AST, ALT, ALP, and γ-GTP were 130 U/L, 120 U/L, 229 U/L, and 163 U/L, respectively, reflecting increases of 5.2-, 10.0-, 2.6-, and 2.7-fold from baseline values (Table 1, Table 2). The prostate-specific antigen level was 16.3 ng/mL, compared with a baseline of 2.4 ng/mL. Abdominal ultrasonography showed no hepatobiliary abnormalities. The patient was diagnosed with acute bacterial prostatitis and prescribed levofloxacin 500 mg daily for 10 days, along with a single dose of loxoprofen. Symptoms gradually improved, and liver function returned to normal within 26 days (Figure 1). Tests for antinuclear, anti-smooth muscle, and anti-mitochondrial M2 antibodies were all negative.

**Figure 2:**
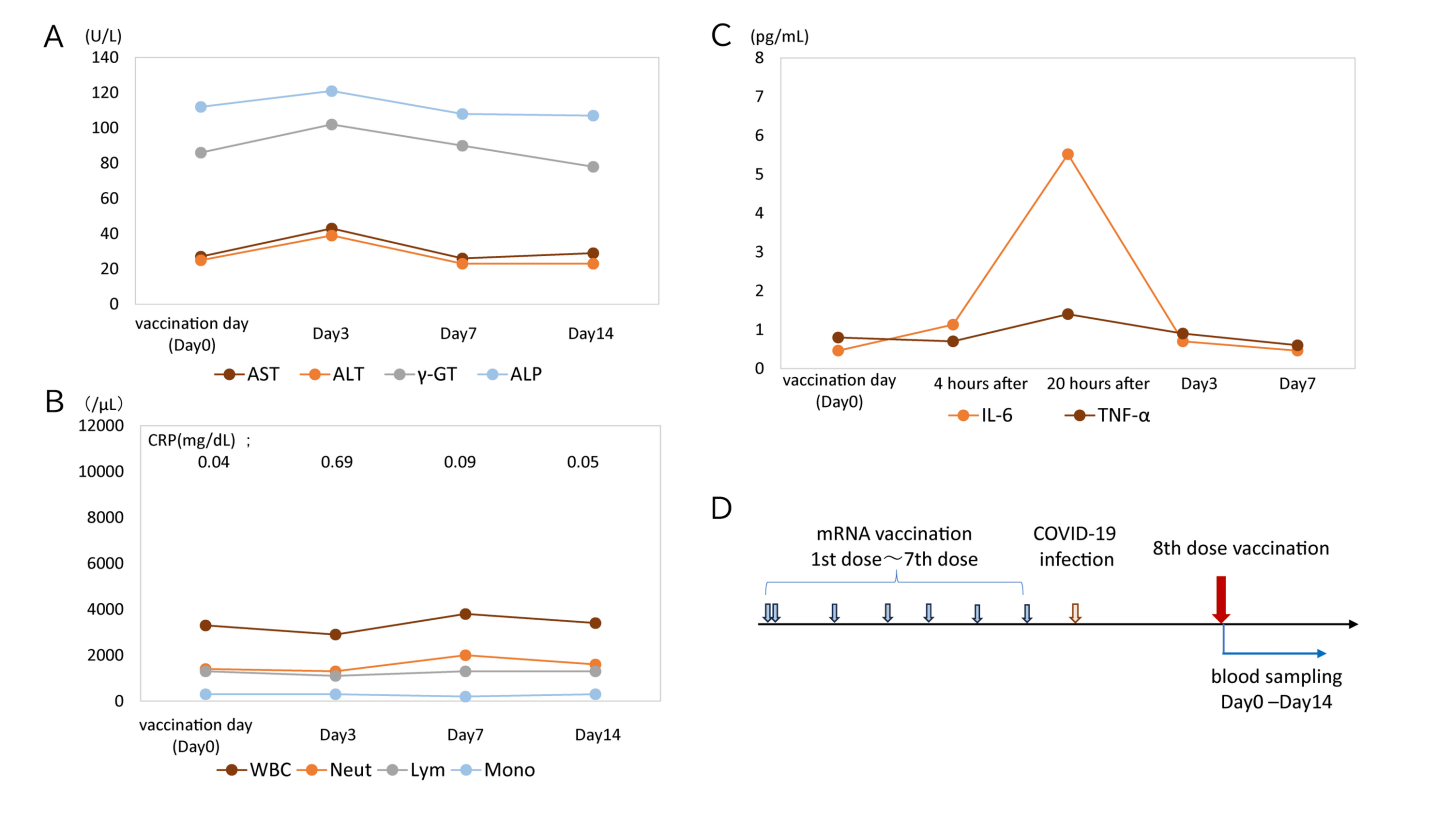
Dynamics of biomarkers before and after the eighth COVID-19 vaccination (A, B) Changes in liver function and peripheral blood parameters at baseline and three, seven, and 14 days post-vaccination. (C) Variations in IL-6 and TNF-α at four hours, 20 hours, three days, and seven days following vaccination. (D) Timeline from the initial COVID-19 vaccination through the seventh vaccination, subsequent COVID-19 infection, and the eighth vaccination. The temporal correlation between the transient IL-6 peak and the subsequent mild elevation of liver enzymes provides clinical evidence supporting a cytokine-mediated mechanism for the observed hepatic injury. ALP, alkaline phosphatase; ALT, alanine aminotransferase; AST, aspartate aminotransferase; γ-GTP, γ-glutamyl transpeptidase; Lym, lymphocyte; Mono, monocyte; Neut, neutrophil; TNF-α, tumor necrosis factor alpha

In October 2025, the patient received the eighth dose of the mRNA vaccine (Episode 3). Cytokine levels, specifically IL-6 and TNF-α, along with blood counts and liver function tests, were measured at baseline, four hours, 20 hours, three days, and seven days post-vaccination. IL-6 and TNF-α peaked at 20 hours post-vaccination, reaching 5.52 pg/mL and 1.4 pg/mL, respectively, and returned to baseline by Day 3 (Figure 2). On Day 3, liver function tests showed elevations in AST (43 U/L), ALT (39 U/L), and γ-GTP (102 U/L), corresponding to 1.7-, 2.1-, and 1.4-fold increases from baseline (Table 1, Table 2). These levels normalized by Day 7. CRP was slightly elevated at 0.69 mg/dL on Day 3 (Figure 1). Systemic symptoms resolved by Day 2.

## Discussion

This case report documents a notable pattern of transient, mild liver injury with similar kinetics observed in a single patient across three distinct inflammatory events: COVID-19, acute prostatitis, and vaccination with an mRNA-based vaccine. Although the parallel progression suggests a potential common inflammatory mechanism, several key limitations must be considered. First, this is a single-case observation, which precludes generalization. Furthermore, cytokine levels were measured only during the vaccination episode; therefore, while proinflammatory cytokines are suspected to be involved, a definitive causal link between liver injury and the preceding infectious episodes cannot be established. Notwithstanding these limitations, the longitudinal nature of this case provides a valuable opportunity to explore potential pathophysiology.

Hepatic injury in patients with COVID-19 has been documented, with a cumulative prevalence of acute liver injury of 22.8%. Men with low lymphocyte levels are at an increased risk of developing this condition [8]. Severe hepatic injury occurs in only 6.4% of patients and is predominantly associated with pre-existing medical conditions [1]. Liver injury in COVID-19 patients can be classified as “hepatocellular type,” in which AST and/or ALT levels exceed three times the upper limit of normal (ULN), or “cholestatic type,” in which ALP levels are elevated to more than twice the ULN [9]. Although this case is consistent with the “cholestatic type,” the pattern of liver injury in COVID-19 patients may not always be definitively categorized.

COVID-19 presents a spectrum of clinical manifestations, ranging from asymptomatic or mild symptoms to severe hyperinflammatory states driven by proinflammatory cytokines, which can progress to acute respiratory distress syndrome and death [2]. The most severe manifestation, known as a CS, is characterized by elevated levels of IL-6, IL-10, and other cytokines, along with severe CD4 and CD8 T-cell lymphopenia and coagulopathy [3]. Compared with patients with nonsevere COVID-19, individuals with severe COVID-19 exhibit significantly higher circulating IL-6 levels (median = 18.63 pg/mL, 95% CI: 10.91-26.35, P < 0.001) [3].

Hepatic injury in COVID-19 patients is attributed to multiple factors, including direct hepatocyte invasion by SARS-CoV-2 via angiotensin-converting enzyme 2, cytokine-mediated liver injury, hypoxic reperfusion injury, drug-induced hepatotoxicity from lopinavir and ritonavir, immune-mediated inflammation, and coagulopathy [10]. In addition to these established mechanisms, other potential contributors, such as subclinical hypoxia or transient metabolic stress, warrant consideration, particularly in milder cases. Even mild inflammatory states may cause subtle alterations in hepatic perfusion or increased metabolic demands on hepatocytes, potentially leading to transient elevations in liver enzymes and reflecting the liver’s sensitivity to systemic physiological changes beyond direct inflammation. However, the cellular and molecular mechanisms underlying COVID-19-related liver injury remain unclear. In this case, COVID-19 led to mild acute liver injury. Although liver injury due to molnupiravir cannot be excluded, the condition remained mild and resolved successfully after treatment.

Acute prostatitis is an acute bacterial infection of the prostate gland, predominantly caused by gram-negative bacilli such as *Escherichia coli*. Clinical manifestations include fever, dysuria, perineal pain, and general malaise, with potential progression to CS and sepsis in severe cases [11]. Cytokines, particularly TNF-α and IL-6, contribute to hepatocellular and sinusoidal endothelial cell injury. The liver plays a critical role in synthesizing and detoxifying acute-phase proteins, and excessive cytokine stimulation may result in elevated liver enzymes, jaundice, and multi-organ damage [4].

Patients with acute bacterial prostatitis are typically treated with oral antibiotics. However, the global spread of multidrug-resistant *E. coli* has rendered fluoroquinolone therapy ineffective in many cases. It is hypothesized that the acute prostatitis in this case was caused by *E. coli*, yet the strain was likely not multidrug-resistant, and fluoroquinolone therapy was effective [11]. The patient received a single oral tablet of levofloxacin on the day preceding the confirmation of liver injury, followed by daily administration for 10 days. Liver enzyme levels subsequently improved, effectively excluding DILI.

On the third day following mRNA vaccination, a transient, mild elevation of AST and ALT was observed, reaching 43 U/L and 39 U/L, respectively. These levels returned to baseline by Day 7. The elevations were modest, measuring 1.7- and 2.1-fold increases from pre-vaccination levels, but were distinct outliers compared with health checkup data from the past decade. A large self-controlled case series in Hong Kong involving over 2.2 million individuals demonstrated that vaccination with BNT162b2 or CoronaVac was not associated with an increased risk of acute liver injury, defined as ALT and AST exceeding three times the ULN and total bilirubin exceeding twice the ULN [12].

Nonetheless, immune-mediated [6] and severe liver injury [13] following COVID-19 vaccination have been reported. In a study of 59 individuals who developed acute liver injury after SARS-CoV-2 vaccination [14], histology revealed predominantly lobular hepatitis without fibrosis. Among these patients, 65% were antinuclear antibody-positive, 54% were anti-smooth muscle antibody-positive, and 35% had elevated IgG levels. Clinical and histological features resembled autoimmune hepatitis, and patients responded well to corticosteroid therapy. Immunological evaluation did not reveal evidence of autoimmune hepatitis or primary sclerosing cholangitis. However, the mechanisms underlying liver injury following COVID-19 vaccination and the relationship between vaccines and the onset of autoimmune disease remain unclear.

A report on cytokine dynamics following COVID-19 mRNA vaccination [5] indicates that both the efficacy and side effects of the vaccine are dependent on immune responses, including proinflammatory cytokine production and lymphocyte activation. IL-6 levels were elevated one day after the first vaccine dose compared with pre-vaccination levels and increased further following the second dose. In contrast, TNF-α levels did not rise after the first dose but showed a significant increase after the second dose. Notably, no correlation was observed between IL-6 and TNF-α levels before vaccination [5]. Local side effects, such as pain and swelling at the injection site, showed a weaker correlation with systemic symptoms, such as fever and myalgia, whereas serum TNF-α levels were associated with systemic adverse reactions [5]. In this case, IL-6 exhibited a pronounced peak 20 hours after mRNA vaccination, reaching a level comparable to previously reported values [5], while TNF-α showed only a marginal increase. Transient liver injury following mRNA vaccination demonstrates considerable interindividual variability and may influence immune responses. Therefore, large-scale prospective cohort studies are needed to further evaluate the impact of these vaccines.

Liver injury has also been associated with respiratory viral infections, including Epstein-Barr virus [15], influenza virus [16], adenovirus [17], coxsackievirus [18], and echovirus [19]. Mild liver injury may occur in primary care settings, where common cold syndromes are frequently treated. The pathophysiology of infectious disease-associated liver injury is multifactorial and remains poorly understood. In this case, similar transient liver injury was observed in response to a virus (COVID-19), bacteria (acute prostatitis), and vaccine (SARS-CoV-2 mRNA), suggesting a proinflammatory cytokine-mediated mechanism. Given the rarity of mild cytokine-induced liver injury, host-specific sensitivity to cytokines may contribute. Genetic polymorphisms in IL-6 and IL-1 have been proposed to influence liver disease progression and risk [20]; however, the underlying molecular mechanisms remain poorly understood.

Serious conditions associated with cytokine release include CS [3] and cytokine release syndrome (CRS), a known adverse event associated with CAR-T cell therapy [21]. The term “cytokine storm” does not denote a specific medical diagnosis but rather a clinical phenomenon characterized by an excessive and uncontrolled immune response, leading to severe systemic inflammation and potential multi-organ failure [3]. Mild cases, such as the one described here, do not fit the full clinical picture of CS and are often overlooked.

The severity of CRS is assessed based on fever, blood pressure, oxygen saturation, and organ toxicity [21]. Grade 1 (mild) CRS is defined as fever alone, with a temperature of ≥38°C, excluding cases with only low-grade fever, such as this patient. This mild presentation, which does not meet the criteria for a CS or CRS, may be more appropriately described as a “cytokine spritz.”

The infrequency of mild cytokine-induced liver injury suggests that host-specific sensitivity to cytokines may play a role. Although genetic polymorphisms in cytokine-related genes have been proposed to influence liver disease, the precise mechanisms remain unclear. Mild, transient liver injury, potentially mediated by low-level cytokine release, is likely under-recognized in primary care. This unique longitudinal case highlights a potential host-specific predisposition to such injuries and underscores the need for further investigation.

## Conclusions

The author reports his own case of recurrent, transient, mild liver injury with similar kinetics following three distinct inflammatory triggers: COVID-19, acute prostatitis, and mRNA vaccination. These observations suggest a potential host-specific predisposition to mild cytokine-mediated organ injury in response to common inflammatory stimuli. Large-scale prospective studies are warranted to assess cytokine profiles during mild infections and vaccinations to further elucidate the pathophysiology and clinical significance of this under-recognized phenomenon.
